# Educating, Contextualizing, and Deferring: Qualitative Investigation of Physician Communication About Chronic Kidney Disease

**DOI:** 10.3390/healthcare14101403

**Published:** 2026-05-20

**Authors:** Amanda Ziegler, Kennedy Walcott-George, Adam Sullivan, Mary Gailor, Liise Kayler, Laurene Tumiel Berhalter

**Affiliations:** 1 Department of Family Medicine, University at Buffalo, Buffalo, NY 14203, USAtumiel@buffalo.edu (L.T.B.); 2 Department of Community Health and Health Behavior, University at Buffalo, Buffalo, NY 14214, USA; 3 Department of Biological Sciences, University at Buffalo, Buffalo, NY 14260, USA; 4 Erie County Medical Center’s Regional Transplantation and Kidney Care Center of Excellence, Department of Surgery, University at Buffalo, Buffalo, NY 14203, USA; liisekay@buffalo.edu; 5 Department of Family Medicine, The Clinical and Translational Science Institute, University at Buffalo, Buffalo, NY 14203, USA

**Keywords:** chronic kidney disease, patient-centered care, physician communication

## Abstract

Background/Objectives: Chronic Kidney Disease (CKD) is a prevalent condition requiring ongoing patient counseling and engagement, yet little is known about how physicians communicate with patients about CKD in routine clinical practice. We conducted a qualitative study to examine physician communication approaches related to CKD and to assess how these approaches align with Picker’s principles of patient-centered care framework. Methods: Semi-structured interviews were conducted with primary care physicians and nephrologists practicing in community and safety-net settings. Using directed content analysis, we identified patterns in how clinicians describe educating patients, contextualizing clinical information, and deferring aspects of counseling to other providers. Results: Physicians predominantly emphasized information-giving and the use of laboratory data to explain disease status. In contrast, practices such as explicit patient preference elicitation, addressing fear, anxiety, or physical comfort, and involving family or support persons were infrequently described. Mapping these communication behaviors to patient-centered care principles highlighted specific elements that are routinely enacted and others that remain underutilized in everyday CKD counseling. Conclusions: These findings identify concrete, feasible opportunities to strengthen patient-centered communication through brief, practice-ready strategies such as plain-language explanations, teach-back, values checks, and shared decision-making prompts. Enhancing these communication practices represents a pragmatic opportunity to improve the quality and patient-centeredness of CKD care.

## 1. Background

Chronic Kidney Disease (CKD) impacts 1 in 7 US adults [1,2]. Patients with advancing CKD experience declining quality of life [3], requiring frequent monitoring and transitions of care from a primary care provider (PCP) to a nephrologist [4] in more advanced disease stages 3 through 5. This creates a high burden for both the patient and the American healthcare system [5]. Despite the increasing prevalence of CKD in the United States, the quality of kidney care is considered inadequate [6]. CKD care often lacks patient-centeredness; conventional US medicine approaches to patient care have been criticized for insufficiently considering individual patient preference, shared input in decision-making, and the competing needs of patients with many comorbidities [7,8,9].

**Figure 1:**
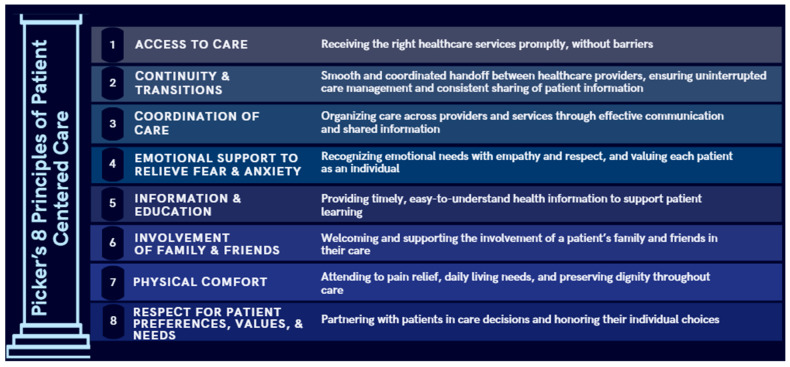
Picker’s 8 principles of patient centered care.

Prioritizing patient-centered approaches may improve the quality of kidney care, as these care models are associated with more positive patient perspectives and improved patient outcomes such as self-efficacy, treatment adherence, and preventive behaviors [10,11,12,13,14]. Picker’s principles framework offers a structured way to evaluate communication founded on respect for patient values and responsiveness to patient needs and preferences and define the core elements necessary to deliver patient centered care [12,15]. The eight principles are outlined and described in Figure 1. Understanding clinicians’ communication approaches with patients is essential because communication is a primary mechanism through which patient-centered care is enacted and through which fears, values, and preferences are elicited. Prior research has used Picker’s principles as a framework to examine patient-centered care for various chronic conditions, including CKD [16,17,18].

Figure 1 Picker’s principles of patient centered care represent eight pillars to ensure that healthcare is patient-centric. This figure lists and describes definitions of each principle. This figure was adapted from Kuipers SJ, Nieboer AP, and Cramm JM, 2021 [19].

Prior work focuses on specific communication contexts (e.g., shared decision-making [20], framing of CKD [21]), but little is known about how physicians as a whole communicate about CKD in routine encounters in both the primary care and nephrology settings. Although primary care providers and nephrologists both counsel patients with CKD at different points in disease progression, little is known about how their communication approaches align with key patient-centered principles [9,10,22].

Understanding strategies PCPs and nephrologists use for counseling patients with CKD is a first step in understanding gaps in approach that may significantly engage a patient in their care and can highlight opportunities for changes that enhance engagement across healthcare teams. The purpose of this qualitative study was to learn about PCP– and nephrologist–patient (hereafter referred to as providers) communication approaches for working with people with CKD and examine gaps and opportunities for improving patient-centered CKD care for patients in the United States [15].

## 2. Methods

We conducted a qualitative study to elicit how physicians communicate with patients about CKD in routine care. Eligible participants were board-certified Family Medicine, General Internal Medicine, or Nephrology physicians (MD/DO) actively caring for patients with CKD at Western New York practices that accept Medicaid. The eligibility criterion that providers see at least some Medicaid patients in their practices was set to ensure inclusion of those with a range of patient populations with CKD [23]. Physicians were selected because they routinely explain CKD concepts and laboratory findings, discuss disease trajectory and treatment options, and address patient questions at varying stages of illness. Including both primary care providers (PCPs) and nephrologists was intended to capture a range of communication approaches across roles; any specialty-based patterns are reported descriptively rather than as formal comparisons. Convenience sampling included sending a study recruitment flyer to local medical professional listservs and direct phone or email advertisements to practices for all local nephrologists within 100 miles of the University. To minimize burden, interviews were conducted via Zoom (San Jose, CA, USA), with verbal consent obtained before recording [24].

A PhD-trained qualitative researcher facilitated each interview while a health sciences master’s student took notes and asked additional follow-up questions where appropriate. Interviews lasted in the range of 30–60 min and used a semi-structured interview guide, with open-ended questions asking about communication approaches providers used with their patients with CKD throughout disease progression. Interview questions differed slightly between provider groups, reflecting the nature of their roles in CKD care. At the end of the interview, we collected participant demographics (e.g., racial identity, years in practice) and practice characteristics (e.g., safety-net insurance proportion, racial/ethnic composition, non-English-speaking patient proportion) and participants received a USD 200 honorarium to help offset their time commitment for the interview.

After each interview, the team debriefed and completed progress notes. Zoom-generated transcripts were reviewed for accuracy by a research assistant, with audio being consulted for clarification. Data collection continued until saturation, defined as no substantive new themes emerging in successive interviews. All procedures were approved by the University’s Institutional Review Board.

### Analysis

All transcripts were de-identified prior to analysis. To support an accelerated analytic timeline while maintaining analytic rigor, each interview was distilled into a 2–3-page summary across pre-specified domains for PCP and nephrologist interviews (e.g., how CKD concepts and laboratory results were explained; how fears, questions, and treatment options were addressed) [24,25,26]. Domains were pre-determined for both the nephrology and PCP interviews based on questions of interest. After each interview, a team member who did not conduct the interview prepared the summary by reviewing the transcript and audio. Next, each summary was reviewed in teams made up of the interviewer, note-takers, and researcher preparing the interview summary. The interview summary under each pre-set analytic domain was revised through discussion until consensus was reached [27]. Interviewer and coder bias were not explicitly assessed, though the interview and study lead was a PhD-level researcher with qualitative methods training and expertise in health services research.

A subset of our patient advisory board (individuals with lived CKD experience; *n* = 4), active since 2012, reviewed PCP and nephrologist summaries separately and met monthly during recruitment, data collection, and coding to provide feedback on the clarity and salience of emerging ideas and to foreground patient-relevant concerns. Patients agreed that the summaries reflected their experiences, noting many troubling miscommunications and missed opportunities in their provider’s approaches where they felt their diagnosis or care plans were not clearly described to them. This input led the research team to consider mapping the rapid qualitative analysis against patient-centeredness principles. This patient advisory board’s input and expertise were critical for ensuring patient voices were considered in research that interviewed providers exclusively [28,29].

For this analysis, only provider approach pre-set domains were analyzed. We conducted a directed content analysis in Atlas.ti (Berlin, Germany; version 25), focusing on communication approaches in relation to Picker’s eight principles of patient-centered care [15]. An initial codebook was developed from four summaries and refined iteratively through team consensus as additional summaries were coded [30]. Coding was organized by participant ID and provider type; any specialty-based patterns were treated as descriptive rather than inferential. For reporting, we characterized dominance of each concept by how often they were generally described and the proportion of providers that mentioned the concept at least once. The presented descriptions are qualitative in nature and are not formal inferential comparisons. Analytic decisions were documented through debriefs and progress notes, and this study followed COREQ guidelines [31]. De-identified data are stored in controlled-access cloud systems at the University at Buffalo; aggregated interview summaries or de-identified coded data are available from the corresponding authors upon reasonable request.

## 3. Results

Findings reflect a range of physician communication strategies used when counseling patients about CKD. These approaches matter because they shape patients’ understanding of CKD, readiness to engage in care, and experiences during ongoing management throughout the healthcare system.

### 3.1. Participant Characteristics

Interview participants included 21 physicians: 7 nephrologists and 14 PCPs. Professional experience ranged across participants, with 43% of each group practicing for 15 years or less. As shown in Table 1, most providers worked with a racially/ethnically diverse patient panel reflecting the local census distribution [32]. About half of providers practiced in settings where few patients qualify for New York State Medicaid insurance, yet several providers served a safety-net sample with high Medicaid utilization. Most providers reported <15% non-English-speaking patients, while two PCPs and one nephrologist had panels with up to 60% non-English speakers. This mix indicates that responses were drawn from a range of practice settings, from affluent suburban practices to those working within more equity-relevant contexts (with high safety-net coverage, language diversity).

Table 1 shows the number of years in practice as well as descriptive details about the interviewed physician’s patient panels in terms of insurance type, racial–ethnic background, and primary language.

### 3.2. Provider Communication Themes

Three themes represented common approaches to patient communication: providing information and education, providing health context, and deferring to other communicators. Patterns sometimes varied by specialty and practice context; descriptions below characterize these descriptively.

#### 3.2.1. Providing Information and Education

Providers reported educating patients about CKD manifestation, disease management, risk factors, and treatment options. Many descriptions reflected primarily information-giving, with fewer examples of structured techniques to elicit preferences or support two-way dialogue. Several providers emphasized the importance of early education as part of continuity of care. PCPs commonly noted engaging patients in decisions (e.g., listing pros/cons, reflecting on personal values, promoting patient ownership). PCPs also more often described addressing fear and anxiety about dialysis, either by projecting a timeline for potential initiation or dispelling misconceptions. As one PCP said, “I’m just available mostly to answer their questions if they’re scared when they do the dialysis.”

Providers also acknowledged barriers that can shape the effectiveness of education, including health literacy, fear, trust in the healthcare system, social support, language, financial constraints, and comorbidities. PCPs frequently described aligning education with patient values, preferences, and expressed needs (e.g., planning timelines, connecting to social resources): “The situation where I would be involved in more is if somebody has any sort of social barriers… transportation is such a huge thing with dialysis because of just the frequency.” Nephrologists commonly discussed barriers such as patient health status, engagement with care, and fear; yet fewer references were made to socioeconomic factors in shaping counseling approaches.

To promote understanding, many providers intentionally used plain language and analogies (e.g., kidneys as a “filter” that clears “toxins and extra fluids”). Some avoided terms like “chronic disease,” opting for phrases such as “kidneys working slowly.” Nephrologists described using non-traditional aids (hand gestures, diagrams/pictures of dialyzers) and directing patients to the National Kidney Foundation website for patient-friendly information.

#### 3.2.2. Providing Health Context

Providers often contextualized information by referencing historical laboratory trends or comparing results to other risk indicators, describing history as “the best predictor of the future.” Personalized health information—risk factors, prior labs, and comorbidities—was used so patients “have a good feeling of where they are, where they were in the past, and also, to discuss with them where things may be heading.” Several PCPs highlighted building baseline understanding through ongoing continuity, discussing kidney-related labs at regular intervals even before a CKD diagnosis (e.g., “today we’re going to check your liver and kidney function and your A1C”). Across specialties, lab trends were described as a predictive tool that can reduce fear about progression or dialysis when interpreted in context and used to time further evaluation.

#### 3.2.3. Deferring to Other Providers

Both specialties described deferring aspects of counseling to other team members. PCPs commonly cited limited depth in CKD subspecialty knowledge as a reason to defer more detailed education to nephrologists or other specialists. As one PCP reflected, “I don’t necessarily need an in-depth knowledge of transplant of kidneys, because that’s what we have the nephrologist for… I have familiarity with it but certainly not to the extent that a nephrologist would.” Some providers noted that when patients were already adherent to medication plans, additional treatment discussions were less emphasized. Several clinicians mentioned limited familiarity with transplant eligibility criteria as a reason to defer advanced treatment conversations. Nephrologists frequently described relying on tertiary services (dialysis and transplant centers) to conduct evaluations and education in advanced CKD, with one noting: “So, transplant the thing is, I don’t provide too much information… I definitely mention [there are] a few hoops… Beyond that I say that I’m going to send your paperwork to [*local transplant hospitals].”

### 3.3. Provider Communication Approaches and Patient-Centered Care Mapping

Table 2 maps observed approaches to Picker’s eight principles of patient-centered care [15]. The predominant emphasis on information and education aligns directly with that principle, with execution differing somewhat across provider roles. The principle of emotional support appeared in efforts to allay dialysis-related fears, which was described more frequently by PCPs in these interviews, while respect for patient values, preferences, and expressed needs emerged where clinicians engaged patients in decision reflections and acknowledged social barriers related to access to care. In contrast, involvement of family and friends and attention to physical comfort were never or rarely described. Both provider groups frequently mentioned practices consistent with continuity and transitions (e.g., using historical labs; rapport building over time). Finally, although relying on others to educate and counsel can detract from a single clinician’s ability to deliver patient-centered care in the moment, such deferral may reflect or require coordination of care, depending on how teams organize responsibilities.

Table 2 organizes the main findings of the qualitative interview analyses related to provider communication approaches with CKD patients across the 8 principles of patient-centered care with an emphasis on identified gaps for each provider type.

Table 3 organizes approaches from Picker’s eight principles to direct quotations from providers.

## 4. Discussion

Across interviews, physicians most often relied on information-giving and laboratory context when discussing CKD, with less consistent use of patient-centered practices such as explicit preference elicitation, addressing fear/anxiety, and involving family or support persons. Mapping these behaviors to Picker’s principles clarified which elements are routinely enacted (information/education; aspects of continuity) and which are under-used (emotional support, respect for values through active preference–elicitation, family/support involvement, attention to physical comfort).

### 4.1. Information/Education and Use of Context

Providers report they predominantly educate their patients by *providing information and education* and they rely on *continuity* of care with their patients to educate effectively. Studies have shown that patient knowledge and comfort about CKD management can be improved by incorporating handouts and multimedia [33,34]. This aligns with our findings that nephrologists attempt to incorporate alternate resources (ex. visuals, handouts, and directing patients to national foundation websites) to augment education of complex information and to facilitate patient understanding. PCPs may benefit from diversifying their educational approach by incorporating other educational modalities into patient counseling thereby improving the patient-centeredness of their care [35,36]. Providers often include contextual health information in their patient communication, by discussing patient health history and laboratory trends. The use of personalized communication increases patient-centeredness and may strengthen the patient–provider relationship [37,38]. These patterns underscore that effective CKD counseling benefits from continuity, ongoing dialogue that tracks trajectory and primes patients for future decisions, congruent with work linking primary-care continuity to better comorbidity management and less acute care among CKD patients [39,40]. Therefore, CKD patients may benefit further from public health efforts that support continuity between providers, rather than alternating providers during repeated clinic visits.

### 4.2. Respect for Values and Emotional Support

Several clinicians described efforts to reduce fear/anxiety (e.g., clarifying dialysis timelines, dispelling misconceptions), yet brief, one-way updates sometimes appeared to replace two-way value elicitation, which can limit respect for patients’ preferences and needs. Providers described approaches to relieve patient fear and anxiety when discussing lab results in a direct response to dispelling patient fears about not imminently needing dialysis treatments, however providers also described actively limiting the information presented to patients and kept descriptions brief, to not alarm patients or cause undue stress [41]. Our interviews suggested potential value in introducing shared decision-making (SDM) techniques earlier in the disease course to align management with what matters to patients and introducing provider-accountability strategies to help ensure that SDM is applied during routine practice [42]. PCP narratives more commonly incorporated social determinants of health (SDOH), health literacy, language, transportation, and cost, where nephrologists’ narrative lacked a description of SDOH. Consistent with calls for clinicians to better understand patients’ individual social and economic circumstances, our interviews suggest that while some providers recognize these needs, additional system-level strategies are necessary to address social barriers earlier and help prevent CKD progression. Providing clinicians with clear, actionable mechanisms to identify and respond to patients’ social needs may strengthen their ability to deliver patient-centered CKD care [43,44].

### 4.3. Coordination/Deferral and Access Trade-Offs

One of the most salient findings of this analysis was providers’ frequent deferral of CKD-related communication to other clinicians, services, or care settings, including nephrologists, dialysis centers, transplant teams, or ancillary staff. When deferral functions well for specific clinical needs, it can reflect coordination of care and support patients in receiving the intended guidance [45]. However, over reliance on deferrals may impose excessive access burdens (additional travel, costs, scheduling), especially for resource-constrained patients [46] and communication gaps can emerge without deliberate closed-loop communication tools [47,48]. Rather than reflecting a simple communication omission, this deferral pattern can be understood as a communication strategy operating within fragmented care pathways [49].

In the US healthcare system, chronic disease management is distributed across multiple providers, settings, and timepoints. Prior work has shown that such care fragmentation is common in chronic disease care and can undermine patients’ understanding of illness, continuity, and trust when coordination is poor [50]. Within this context, deferral may function as a form of role delineation, whereby physicians intentionally limit discussion of topics perceived to fall outside their expertise, formal responsibility, or available time [51]. From this perspective, deferral can be adaptive when it reflects clear role boundaries, effective interprofessional communication, and reliable downstream follow-through [52]. For example, deferring detailed dialysis or transplant counseling to specialized, structured education teams may allow patients to receive more accurate and comprehensive information, particularly when accompanied by transparent explanation and anticipatory guidance. In such cases, deferral may support coordination by aligning communication with clinical expertise [52].

However, our findings suggest that deferrals may also become problematic under conditions of fragmented care, role ambiguity, or limited coordination. These findings position deferral not simply as a missing behavior, but as a site where structural fragmentation becomes visible at the level of clinician–patient communication. When communication about prognosis, options, or emotional concerns is deferred without explicit framing, or when patients are expected to navigate referrals independently, deferral may leave gaps in patient understanding, exacerbate anxiety, or undermine patient-centeredness [52]. In these instances, deferral operates less as a coordinated handoff and more as shifting the burden of sense-making to patients. Importantly, distinctions between adaptive and problematic deferral appeared less related to provider specialty than to whether clinicians maintained ownership of communication, even when clinical roles and decision-making responsibilities were shared across the care team [53,54]. Clinician practices such as awareness of their deferring behaviors, naming the reason for deferral to patients, previewing what patients can expect next, and checking for understanding may help mitigate the risks associated with fragmented care pathways. Strengthening patient-centered CKD care may therefore require not only better information delivery, but clearer communication ownership across transitions and roles.

### 4.4. Under-Used Elements: Family/Support Involvement and Physical Comfort

Two elements of Picker’s framework were rarely described: involving family/friends and addressing physical comfort. This mirrors reports that pain and comfort needs may be overlooked in CKD decision discussions [55]. Yet involving family or trusted support during chronic-disease visits is associated with more thorough discussions and improved understanding of medical advice [56,57]. Normalizing the presence of a support person, where acceptable to the patient, and adding a brief, routine comfort check could strengthen patient-centeredness without major workflow disruption [58]. In practice, clinicians sometimes rely on social workers or patient navigators to surface and address family and social-support needs and to troubleshoot related concerns, especially for CKD patients on dialysis [58,59,60,61]. While this clinical collaboration is valuable [62,63,64], over-dependence on ancillary roles may leave these elements under-addressed during the provider encounter itself. Brief, physician-delivered prompts (e.g., inviting a support person, asking about pain or treatment-related discomfort) can complement team-based efforts and ensure these patient-centered elements are consistently incorporated into CKD counseling.

It is also important to note that nuances of the Picker’s framework may have various levels of applicability depending on clinical settings. For example, it is possible that CKD care in outpatient settings may not focus on addressing patients’ physical comfort to the extent that a treatment (i.e., dialysis center) or inpatient setting would; therefore, this aspect of the Picker’s framework may not need to be emphasized to the same degree as other aspects in this analysis.

### 4.5. Strengths and Limitations

This study had many strengths. First, the study benefits from a diverse group of physician participants, allowing us to capture communication experiences relevant to patients seen in a variety of practice settings. The use of rigorous qualitative procedures, including team-based consensus review, multiple interviewers to mitigate interviewer bias, iterative refinement of the codebook, and mapping onto established patient-centered care framework strengthens the analysis and findings.

At the same time, several limitations are offered for consideration. Because this study relies on physicians’ self-selection to participate in the study and provision of descriptions of how they communicate rather than direct observation, responses may reflect socially desirable portrayals of clinical interactions and limits the level of interpretation [65]. Additionally, our sample included more PCPs than nephrologists, and while this proportion is in line with the distribution of general versus specialty practitioners in the field, as a result, any specialty-related patterns should be interpreted as descriptive rather than comparative. Our sample also represents the healthcare system of a single region, which may shape physician communication patterns via constraints on visit length, resource scarcity, and reimbursement models. Lastly, our work captured the patient provider communication through the provider’s perspective, without direct input from patients. While a patient advisory board was involved in the creation, analysis, and reporting of the research, we did not have the ability to triangulate patient perspectives alongside provider perspectives in this analysis, and therefore this study only considers the perspectives of physicians about their clinical approaches.

### 4.6. Recommendations

Findings point to several feasible communication supports that clinicians could consider adopting in routine CKD care. These include concise visuals and plain-language scripts for explaining renal function and CKD to better reach patients with different learning styles and lower health literacy [66], as well as routine use of teach-back to confirm patient understanding [67]. To address multiple communication gaps identified in this study, clinicians may also benefit from more consistent use of shared decision-making, including repeated checks on patient values and explicit discussion of treatment choices and preferences during counseling encounters. Such practices respect patient autonomy and place patients at the center of care while simultaneously creating space to surface fear, anxiety, pain, and concerns related to SDOH (e.g., through a brief “What concerns you most right now?” prompt) [68,69,70]. Other recommendations include systematically incorporating SDOH considerations into CKD counseling, through practice-based screening questions paired with direct referral pathways for language, financial, or transportation support. These strategies may help reinforce the access and coordination elements of patient-centered care that are particularly critical given the frequency and complexity of care required as CKD progresses [71,72]. Where feasible, targeted clinical refresher lessons focused on CKD-specific communication may also bolster PCP confidence, potentially reducing unnecessary deferral and early referral burden for nephrology care [73].

## 5. Conclusions

This study clarifies which communication strategies PCPs and nephrologists report using when counseling patients with CKD and identifies specific patient-centered elements that may be inconsistently enacted. Physicians predominantly described information-giving and laboratory value reporting as central components of CKD communication, while some topics were intentionally limited or deferred to other professionals. Together, these patterns reveal missed opportunities to implement the full range of patient-centered communication practices in routine CKD care.

Systematic use of bidirectional communication techniques, including explicit patient preference elicitation, brief assessment of fear, anxiety, and physical comfort, and routine invitation of family or support persons, delivered through brief, practical supports (e.g., shared decision-making prompts, values checks, teach-back, plain-language visuals) may represent pragmatic strategies to strengthening patient-centered CKD counseling for both PCP and nephrologists. Future research should incorporate patient-reported experience measures or brief observational approaches to triangulate clinician-reported communication behaviors with patient experiences and should evaluate the acceptability and impact of lightweight communication tools across diverse practice settings.

## Figures and Tables

**Table 1:** Participant demographics and patient panel estimates.

	Primary Care Physicians *n* (%) (*n* = 14)	Nephrologists *n* (%) (*n* = 7)	Total *n* (%) (*n* = 21)
Physician Years in practice			
0–15	6 (43)	3 (43)	9 (43)
16–45	8 (57)	4 (57)	12 (57)
% Medicaid insured patient panel			
0–25%	8 (57)	2 (29)	10 (48)
26–50%	3 (21)	3 (43)	6 (29)
51–100%	3 (21)	0 (0)	3 (14)
Unknown	0 (0)	2 (29)	2 (10)
% Black/African American patient panel			
0–25%	12 (86)	3 (43)	15 (71)
26–50%	2 (14)	3 (43)	5 (24)
51–75%	0 (0)	1 (14)	1 (5)
% Hispanic/Latino patient panel			
0–5%	7 (50)	3 (43)	10 (48)
6–10%	6 (43)	2 (29)	8 (38)
11–20%	1 (7)	2 (28)	3 (14)
% Non-English-speaking patient panel			
0–15%	12 (86)	5 (71)	17 (80)
16–30%	0(0)	1 (14)	1 (5)
45–60%	2 (14)	0 (0)	2 (10)
Unknown	0 (0)	1 (14)	1 (5)

**Table 2:** Mapping of patient-centered communication approaches to identify gaps in care communication.

Principle	PCP Communication Approach	Nephrologist Communication Approach	Identified Gaps
Access to care	Considers barriers that may impact access to care (language, transportation, insurance, etc.) Considers resources necessary for viability of treatment options (in-home dialysis, transportation lifestyle challenges)	Considers resources necessary for viability of treatment options (in-home dialysis, transportation lifestyle challenges)	Improve PCPs’ and nephrologists’ understanding of dialysis and transplant requirements to support patient’s access PCPs’ and nephrologists can assess barriers created by SDOH, and better link patients to services
Care continuity and transitions	Uses historical lab results to educate patients and make referral decisions Communicates lab results to build health literacy Builds relationships to dispel fears	Uses historical lab results to educate patients and make referral decisions Communicates lab results to build health literacy Builds relationships to dispel fears	Some expressed discomfort referring to transplantation services due to limited physician knowledge and familiarity Refer more regularly to transplant services
Coordination of care	Connects patients to nephrology Encourages/arranges social services for patients (home nurses, transportation, language help, etc.) Connects patients with specialists/resources such as transplant teams, dialysis centers, and CKD educational programs	Connects patients to nephrology Encourages/arranges social services for patients (home nurses, transportation, language help, etc.) Connects patients with specialists/resources such as transplant teams, dialysis centers, and CKD educational programs	Nephrology: consider more 2-way communication with PCPs about patient co-management Nephrology: increase focus on social service coordination that patients may need to participate fully in their care
Emotional support to relieve fear and anxiety	Some express an understanding of barriers to care and life circumstances Limited address of patient’s emotional needs, described as providing reassurance for timeline to needing dialysis	Limited address of patient’s emotional needs, described as providing reassurance for timeline to needing dialysis	PCPs and nephrologists can address patient fear and anxiety more holistically
Information and education	Main way of communicating with patients is through CKD education Some consider patient barriers (language, health literacy, etc.) when providing information and education, but few use teach-backs or methods to verify patient understanding Defers to nephrology or dialysis centers to provide patient information on advanced CKD and treatment options Uses simplified explanations	Main way of communicating with patients is through CKD education Some consider patient barriers (language, health literacy, etc.) when providing information and education, but few use teach-backs or methods to verify patient understanding Defers to nephrology or dialysis centers to provide patient information on advanced CKD and treatment options Uses simplified explanations Some nephrologists use alternative resources like diagrams, gestures, and patient-centered websites	PCPs could use alternative resources for patient education to accommodate various levels of health literacy and learning styles PCPs could bolster their CKD and renal knowledge to better support patient education at earlier stages Nephrology may consider screening for or discussing SDOH with patients as part of care plan co-development
Involvement of family and friends	Not widely discussed as a communication strategy	Not widely discussed as a communication strategy Recognizes that family and friends can be outside influences on patient’s treatment choice	Incorporate discussion of social support in conversations with CKD patients
Physical comfort	Not discussed as a communication strategy or care management focus area	Not discussed as a communication strategy or care management focus area	Address physical comfort and pain with patients when exploring treatment options
Respect for patient values, preferences, and expressed needs	Includes patient value systems in decisions about treatment options Utilizes alternate communication styles to meet patient needs Connects patients to supportive resources	Connects patients to supportive resources	Lack of strategies to engage patient value systems in nephrology

**Table 3:** Patient-centered communication approaches exemplified by physicians’ quotations.

Principle	Supporting Quotation(s)
Access to care	“From a social issue standpoint, transportation is such a huge thing with dialysis because of just the frequency. A lot of patients report to me that they end up having some difficulty with Medicare cabs or Medicaid cabs… So sometimes my office will help with that”—PCP14
Care continuity and transitions	“In terms of alleviating anxiety…I’m able to alleviate their anxiety fairly easily in the patients who need attention, who need say dialysis in the next 2, 3 years. I mean, these patients are being seen by me serially so during their follow up, even if I’m not able to educate them… as time progresses, I am able to”—Neph4
Coordination of care	“So, we do have some like pre-ESRD education classes…We try to engage the nurses, social workers, people like that to kind of continue having the discussions. And then we give patients the number for the transplant center to go over to start to meet with the transplant center”—Neph7
Emotional support to relieve fear and anxiety	“I think when I put it in a percentage that makes it a little bit more easier for them to understand what’s going on, and usually they get kind of scared…and they think the worst-I’m gonna end up on dialysis. So I tell them…people generally don’t go on dialysis until their GFR hits 10 and they’re 45 and they’re not that close and don’t worry”—PCP17
Information and education	“If I can get a handout that I can find. Sometimes I will do that, or I will direct them to websites that I feel can give them good general overview.”—Neph2 “The health literacy here is quite low. So trying to explain renal function in terms that patients understand can sometimes be quite challenging.”—PCP21
Involvement of family and friends	“But usually by the time they’re seeing me, someone has told them that they know someone on dialysis or do they need a kidney transplant. Those ideas are baked in their conscious when we have the meeting”—Neph6
Physical comfort	Not discussed as a communication strategy or care management focus area
Respect for patient values, preferences, and expressed needs	“Then it becomes a question of well, you know, how does getting dialysis relate to your personal value system and what you find important? And how does it relate to your quality of life… and where do we go from here?”—PCP14

## Data Availability

The transcripts used to analyze the results of this study are not currently available due to participant privacy, but are available from the corresponding authors on reasonable request.

## Abbreviations

CKD	Chronic Kidney Disease
PCP	Primary Care Physician
SDM	Shared Decision-Making
SDOH	Social Determinants of Health

## References

[1] Medicine J.H. End Stage Renal Disease (ESRD). https://www.hopkinsmedicine.org/health/conditions-and-diseases/end-stage-renal-failure.

[2] CDC Chronic Kidney Disease in the United States. https://www.cdc.gov/kidney-disease/php/data-research/index.html.

[3] Cruz M.C., Andrade C., Urrutia M., Draibe S., Nogueira-Martins L.A., Sesso Rde C. (2011). Quality of life in patients with chronic kidney disease. Clinics.

[4] Fraser S.D., Roderick P.J., May C.R., McIntyre N., McIntyre C., Fluck R.J., Shardlow A., Taal M.W. (2015). The burden of comorbidity in people with chronic kidney disease stage 3: A cohort study. BMC Nephrol..

[5] Weiner D.E. (2009). Public health consequences of chronic kidney disease. Clin. Pharmacol. Ther..

[6] Tummalapalli S.L., Powe N.R., Keyhani S. (2019). Trends in Quality of Care for Patients with CKD in the United States. Clin. J. Am. Soc. Nephrol..

[7] Evidence Based Medicine Working Group (1992). A new approach to teaching the practice of medicine. JAMA.

[8] Green A.R., Carrillo J.E., Betancourt J.R. (2002). Why the disease-based model of medicine fails our patients. West. J. Med..

[9] O’Hare A.M., Rodriguez R.A., Bowling C.B. (2016). Caring for patients with kidney disease: Shifting the paradigm from evidence-based medicine to patient-centered care. Nephrol. Dial. Transpl..

[10] O’Hare A.M. (2018). Patient-Centered Care in Renal Medicine: Five Strategies to Meet the Challenge. Am. J. Kidney Dis..

[11] Epstein R.M., Street R.L. (2011). The values and value of patient-centered care. Ann. Fam. Med..

[12] Ronald M.E., Richard L.S. (2007). Patient-Centered Communication in Cancer Care: Promoting Healing and Reducing Suffering.

[13] Institute of Medicine Committee on Quality of Health Care in America (2001). Crossing the Quality Chasm: A New Health System for the 21st Century.

[14] Stewart M., Brown J.B., Donner A., McWhinney I.R., Oates J., Weston W.W., Jordan J. (2000). The impact of patient-centered care on outcomes. J. Fam. Pract..

[15] Gerteis M., Edgman-Levitan S., Dalley J., Delbanco T. (2002). Through the Patient’s Eyes.

[16] Loban K., Milland T., Hales L., Lam N.N., Dipchand C., Sandal S. (2025). Understanding the Healthcare Needs of Living Kidney Donors Using the Picker Principles of Patient-centered Care: A Scoping Review. Transplantation.

[17] Furmenti M.F., Bertarelli G., Ferrè F. (2025). Person-centred care in oncological home services: A scoping review of patients’ and caregivers’ experience and needs. BMC Health Serv. Res..

[18] Hagendijk M.E., Zipfel N., Melles M., van der Wees P.J., Hulshof C.T.J., Çölkesen E.B., Hoving J.L., van der Burg-Vermeulen S.J. (2025). Towards person-centred work-focused healthcare for people with cardiovascular disease: A qualitative exploration of patients’ experiences and needs. Disabil. Rehabil..

[19] Kuipers S.J., Nieboer A.P., Cramm J.M. (2021). Easier Said Than Done: Healthcare Professionals’ Barriers to the Provision of Patient-Centered Primary Care to Patients with Multimorbidity. Int. J. Environ. Res. Public Health.

[20] Mandel E.I., Fox M., Schell J.O., Cohen R.A. (2024). Shared Decision-Making and Patient Communication in Nephrology Practice. Adv. Kidney Dis. Health.

[21] Tiu H., Fagerlin A., Roney M., Kerr E., Ojo A., Rothman E., Nunes J.W. (2018). Provider perspectives on chronic kidney disease diagnosis delivery. Clin. Nephrol..

[22] Bowling C.B., O’Hare A.M. (2012). Managing older adults with CKD: Individualized versus disease-based approaches. Am. J. Kidney Dis..

[23] Dicicco-Bloom B., Crabtree B.F. (2006). The qualitative research interview. Med. Educ..

[24] Nicholas S.B., Kalantar-Zadeh K., Norris K.C. (2015). Socioeconomic disparities in chronic kidney disease. Adv. Chronic Kidney Dis..

[25] Nevedal A.L., Reardon C.M., Opra Widerquist M.A., Jackson G.L., Cutrona S.L., White B.S., Damschroder L.J. (2021). Rapid versus traditional qualitative analysis using the Consolidated Framework for Implementation Research (CFIR). Implement. Sci..

[26] Gale R.C., Wu J., Erhardt T., Bounthavong M., Reardon C.M., Damschroder L.J., Midboe A.M. (2019). Comparison of rapid vs in-depth qualitative analytic methods from a process evaluation of academic detailing in the Veterans Health Administration. Implement. Sci..

[27] Goulding M., Borg A., Minkah P., Branley C., Desrochers O., Schwartz E., Uribe P., Powers D., Rosal M.C., Lemon S.C. (2025). Lessons Learned in Developing a Model for Academic-Community Partnered Rapid Qualitative Research. Prog. Community Health Partnersh..

[28] Warren M., Leamon T., Hall A., Twells L., Street C., Stordy A., Majumdar K., Breault L., Fiest K., Rasiah J. (2020). The Role of Patient Advisory Councils in Health Research: Lessons From Two Provincial Councils in Canada. J. Patient Exp..

[29] Brockman T.A., Balls-Berry J.E., West I.W., Valdez-Soto M., Albertie M.L., Stephenson N.A., Omar F.M., Moore M., Alemán M., Berry P.A. (2021). Researchers’ experiences working with community advisory boards: How community member feedback impacted the research. J. Clin. Transl. Sci..

[30] Hsieh H.F., Shannon S.E. (2005). Three approaches to qualitative content analysis. Qual. Health Res..

[31] Tong A., Sainsbury P., Craig J. (2007). Consolidated criteria for reporting qualitative research (COREQ): A 32-item checklist for interviews and focus groups. Int. J. Qual. Health Care.

[32] Bureau U.C. Erie County Quick Facts. https://www.census.gov/quickfacts/eriecountynewyork.

[33] John R. (2023). Utilizing an Educational Patient Handout to Increase CKD Knowledge in Primary Care.

[34] Chiou C.P., Chung Y.C. (2012). Effectiveness of multimedia interactive patient education on knowledge, uncertainty and decision-making in patients with end-stage renal disease. J. Clin. Nurs..

[35] Hong Y.R., Jo A., Cardel M., Huo J., Mainous A.G. (2020). Patient-Provider communication with teach-back, patient-centered diabetes care, and diabetes care education. Patient Educ. Couns..

[36] Narva A.S., Norton J.M., Boulware L.E. (2016). Educating Patients about CKD: The Path to Self-Management and Patient-Centered Care. Clin. J. Am. Soc. Nephrol..

[37] Smith K.L., Martini J. (2023). Patient-provider communication and interactions. Chronic Illness Care: Principles and Practice.

[38] Alanzi T.M., Alanzi N., Majrabi A., Alhajri A.S., Alzahrani L., Alqahtani N., Alqadhibi A., Alenazi S., Alsaedi H., Alghamdi E. (2024). Exploring Patient Preferences Related to Shared Decision-Making in Chronic Disease Management. Cureus.

[39] Chong C., Campbell D., Elliott M., Aghajafari F., Ronksley P. (2022). Determining the Association Between Continuity of Primary Care and Acute Care Use in Chronic Kidney Disease: A Retrospective Cohort Study. Ann. Fam. Med..

[40] Khanam M.A., Kitsos A., Stankovich J., Castelino R., Jose M., Peterson G.M., Wimmer B., Razi Zaidi T., Radford J. (2019). Association of continuity of care with blood pressure control in patients with chronic kidney disease and hypertension. Aust. J. Gen. Pract..

[41] Wright Nunes J., Roney M., Kerr E., Ojo A., Fagerlin A. (2016). A diagnosis of chronic kidney disease: Despite fears patients want to know early. Clin. Nephrol..

[42] Muscat D.M., Shepherd H.L., Nutbeam D., Trevena L., McCaffery K.J. (2021). Health Literacy and Shared Decision-making: Exploring the Relationship to Enable Meaningful Patient Engagement in Healthcare. J. Gen. Intern. Med..

[43] Chuang E., Safaeinili N. (2024). Addressing Social Needs in Clinical Settings: Implementation and Impact on Health Care Utilization, Costs, and Integration of Care. Annu. Rev. Public Health.

[44] De Marchis E., Knox M., Hessler D., Willard-Grace R., Olayiwola J.N., Peterson L.E., Grumbach K., Gottlieb L.M. (2019). Physician Burnout and Higher Clinic Capacity to Address Patients’ Social Needs. J. Am. Board. Fam. Med..

[45] Neale E.P., Middleton J., Lambert K. (2020). Barriers and enablers to detection and management of chronic kidney disease in primary healthcare: A systematic review. BMC Nephrol..

[46] Hounkpatin H.O., Leydon G.M., Veighey K., Armstrong K., Santer M., Taal M.W., Annells P., May C., Roderick P.J., Fraser S.D. (2020). Patients’ and kidney care team’s perspectives of treatment burden and capacity in older people with chronic kidney disease: A qualitative study. BMJ Open.

[47] Greer R.C., Liu Y., Cavanaugh K., Diamantidis C.J., Estrella M.M., Sperati C.J., Soman S., Abdel-Kader K., Agrawal V., Plantinga L.C. (2019). Primary Care Physicians’ Perceived Barriers to Nephrology Referral and Co-management of Patients with CKD: A Qualitative Study. J. Gen. Intern. Med..

[48] Patel M.P., Schettini P., O’Leary C.P., Bosworth H.B., Anderson J.B., Shah K.P. (2018). Closing the Referral Loop: An Analysis of Primary Care Referrals to Specialists in a Large Health System. J. Gen. Intern. Med..

[49] Cheng C., Christensen M. (2026). Understanding Nurses’ Experiences of Fragmented Care in Aging Populations: A Meta-Synthesis. J. Nurs. Res..

[50] Joo J.Y. (2023). Fragmented care and chronic illness patient outcomes: A systematic review. Nurs. Open.

[51] Manca D.P., Breault L., Wishart P. (2011). A tale of two cultures: Specialists and generalists sharing the load. Can. Fam. Physician.

[52] Mehrotra A., Forrest C.B., Lin C.Y. (2011). Dropping the baton: Specialty referrals in the United States. Milbank Q..

[53] Kiger M.E., Meyer H.S., Hammond C., Miller K.M., Dickey K.J., Hammond D.V., Varpio L. (2019). Whose Patient Is This? A Scoping Review of Patient Ownership. Acad. Med..

[54] Hirschman K.B., Shaid E., McCauley K., Pauly M.V., Naylor M.D. (2015). Continuity of Care: The Transitional Care Model. Online J. Issues Nurs..

[55] Lambourg E., Colvin L., Guthrie G., Murugan K., Lim M., Walker H., Boon G., Bell S. (2021). The prevalence of pain among patients with chronic kidney disease using systematic review and meta-analysis. Kidney Int..

[56] Patel S.S., Peterson R.A., Kimmel P.L. (2005). The impact of social support on end-stage renal disease. Semin. Dial..

[57] Rosland A.M., Piette J.D., Choi H., Heisler M. (2011). Family and friend participation in primary care visits of patients with diabetes or heart failure: Patient and physician determinants and experiences. Med. Care.

[58] Urbanski M., Browne T., Plantinga L. (2024). The Dialysis Social Worker: Patients’ Best Defense to Meet Health-Related Social Needs. Am. J. Kidney Dis..

[59] Browne T., Merighi J.R., Washington T., Savage T., Shaver C., Holland K. (2019). Nephrology Social Work. Handbook of Health Social Work.

[60] Lederer E., Lebowitz J. (2020). Current State of the Workforce in Nephrology. Adv. Chronic Kidney Dis..

[61] Meran S., Don K., Shah N., Donovan K., Riley S., Phillips A.O. (2011). Impact of chronic kidney disease management in primary care. QJM Int. J. Med..

[62] Arooj H., Aman M., Hashmi M.U., Nasir Z., Zahid M., Abbas J., Amjad N., Maryam S., Farhan K. (2025). The impact of nurse-led care in chronic kidney disease management: A systematic review and meta-analysis. BMC Nurs..

[63] Feryn N., Boeckxstaens P., Ashcroft R., De Corte J., Roose R. (2024). Looking Through the Eyes of General Practitioners: The Role of Social Work in Primary Health Care. Br. J. Soc. Work.

[64] Ashcroft R., Sheffield P., Adamson K., Phelps F., Webber G., Walsh B., Dallaire L.F., Sur D., Kemp C., Rayner J. (2024). Scoping review of social workers’ professional roles in primary care. BMJ Open.

[65] Bispo Júnior J.P. (2022). Social desirability bias in qualitative health research. Rev. Saude Publica.

[66] Wittink H., Oosterhaven J. (2018). Patient education and health literacy. Musculoskelet. Sci. Pract..

[67] Andersen-Hollekim T., Landstad B.J., Solbjør M., Kvangarsnes M., Hole T. (2021). Nephrologists’ experiences with patient participation when long-term dialysis is required. BMC Nephrol..

[68] Stiggelbout A.M., Van der Weijden T., De Wit M.P., Frosch D., Légaré F., Montori V.M., Trevena L., Elwyn G. (2012). Shared decision making: Really putting patients at the centre of healthcare. BMJ.

[69] Elwyn G., Frosch D.L., Kobrin S. (2016). Implementing shared decision-making: Consider all the consequences. Implement. Sci..

[70] Stiggelbout A.M., Pieterse A.H., De Haes J.C. (2015). Shared decision making: Concepts, evidence, and practice. Patient Educ. Couns..

[71] Buitron de la Vega P., Losi S., Sprague Martinez L., Bovell-Ammon A., Garg A., James T., Ewen A.M., Stack M., DeCarvalho H., Sandel M. (2019). Implementing an EHR-based Screening and Referral System to Address Social Determinants of Health in Primary Care. Med. Care.

[72] Adler N., Cutler D., Fielding J., Galea S., Glymour M., Koh H., Satcher D. (2016). Addressing Social Determinants of Health and Health Disparities: A Vital Direction for Health and Health Care. NAM Perspect..

[73] Johnson M.L., Zimmerman L., Welch J.L., Hertzog M., Pozehl B., Plumb T. (2016). Patient activation with knowledge, self-management and confidence in chronic kidney disease. J. Ren. Care.

